# Much Educational Matter Addressed to the Public

**Published:** 1918-06-29

**Authors:** 


### THE CRICHTON ROYAL INSTITUTION.

### Much Educational Matter Addressed to the Public.

We have received the reports of the Crichton Royal Institution for the years 1913, 1914, and 1917, and are pleased to see that they contain a good deal of educational matter addressed to the general public. We do not agree with all the teaching in every case, but it is a good thing that directors of asylums should place their views of insanity before the public, whether the views are right or wrong. It is well that the thing should be discussed, and that the people should become familiar with that aspect of insanity which regards it as a disease comparable in many respects with other diseases, although in some respects peculiar, and not a mysterious dispensation of Providence.

### The Incidence of Insanity.

Dr. Easterbrook tells his readers that no one becomes insane without previously being, or becoming nervous. If by nervous Dr. Easterbrook means that the brain is an unstable brain, or has some morbid quality which does not exist in the brains of healthy people, he is either wholly wrong or he restricts the meaning of the word "insanity" unwarrantably. The insane are not a class apart. Anyone may become insane, however healthy his brain, and however stably constituted his nervous system, provided only that he is subjected to a sufficiently powerful stress. Anyone who receives a sufficient dose of the microbes of smallpox or typhoid fever may become delirious in the course of the disease, and when he is delirious he is insane. The fact that we call insanity occurring in such circumstances by the name of delirium ought not to blind us to the fact that it is still insanity; but alienists more than any other people are misled by names, and think that the nature of the thing can be altered by calling it by another name. Again, we have only to administer to a man of the soundest constitution a sufficient dose of alcohol and he will certainly become insane. The insanity will only be temporary. It will last only until the alcohol is eliminated; but as long as it lasts it will still be insanity, although we say that the man is "only drunk." The proclivity to insanity varies by insensible degrees among different people. Some are so prone to become insane that the least little stress, either bodily or mental, will throw them off their balance. Others are so stably constituted that it requires a very powerful stress to render them insane; but no person is immune from insanity any more than he is immune from influenza, or from breaking his leg.

### "Heredity and Stress."

We are heartily in agreement with Dr. Easterbrook's teaching on the matter of heredity. It does not follow in the least that the offspring of one insane parent, or even of two insane parents, should necessarily be or become insane; nor does it follow that because a person's parents are both of them sane and of healthy constitution their offspring is sane and immune from insanity. Dr. Easterbrook disagrees with the maxim, which is now becoming familiar, that the etiology of insanity may be summed up in the words "heredity and stress," but prefers to say "nervous constitution and stress," and he says that the nervous constitution may be either inherited or acquired; but he misses the point that if the nervous constitution is acquired, it is acquired through the incidence of continued or repeated stresses. He says that morbid heredity does not necessarily deteriorate the progeny wholesale except in exceptional cases. This is, of course, quite true, but the author of the doctrine that the causes of insanity are heredity and stress has never held, at least has never taught, that morbid heredity does necessarily deteriorate the progeny.

In his report for 1914 Dr. Easterbrook makes a strong appeal, with which we are entirely in sympathy, for legislation which should permit the reception of voluntary boarders into public asylums as well as into registered hospitals and licensed houses. The provision is not only just, but it is also calculated to arrest many cases of insanity in the early stages, and to restore the patients to a useful life without the necessity of certification, and without the necessity of prolonged residence in asylums.

### Deterrents to Treatment.

In his report for 1917, the only asylum report for last year which has yet reached us, Dr. Easterbrook emphasises the larger percentage of recoveries among voluntary patients than among certified patients. This, of course, is only to be expected, for voluntary patients exhibit a milder form of insanity than certified patients, and on the whole constitute a class much more likely to recover. He points out that the two deterrents to the early treatment of insanity are the application to the parish and certification as insane, or what may be called the stigma of pauperism and the stigma of insanity. There is no doubt that these two circumstances have a powerful effect in postponing treatment in an institution, and thereby diminishing the chances of recovery. If cases in an early stage, before they are certifiable, could be received into public asylums, there is no doubt that a certain proportion of them would escape altogether the necessity of certification.

### Meteorology and the Study of Insanity.

Dr. Easterbrook appears to be an enthusiastic meteorologist, and has for several years past kept elaborate records of the meteorological conditions that he obtained at Dumfries. We are afraid that these investigations, however they may be correlated with the occurrence and the fluctuations of insanity, will not throw very much light upon the nature of the disease and the conditions which influence it; but the subject is so obscure that the investigations even of the most unpromising description are to be welcomed.

We regret to see that even in these times of shortage of paper so much space is occupied in these reports with the dreary and useless statistical tables. The directors of asylums might very well follow the example of the English Board of Control, which has omitted the publication of statistical tables for the duration of the war. No one pays any attention to these tables, and no one with any sense attaches any importance to them. A summary of the results, containing all that is useful, and probably a good deal more than is reliable, might easily be comprised within the limits of a single page.

